# Stochastic IMT (Insulator-Metal-Transition) Neurons: An Interplay of Thermal and Threshold Noise at Bifurcation

**DOI:** 10.3389/fnins.2018.00210

**Published:** 2018-04-04

**Authors:** Abhinav Parihar, Matthew Jerry, Suman Datta, Arijit Raychowdhury

**Affiliations:** ^1^School of Electrical and Computer Engineering, Georgia Institute of Technology, Atlanta, GA, United States; ^2^Department of Electrical Engineering, University of Notre Dame, Notre Dame, IN, United States

**Keywords:** stochastic neuron, insulator-metal transition, FitzHugh-Nagumo (FHN) neuron model, Ornstein-Uhlenbeck process, threshold noise, vanadium-dioxide

## Abstract

Artificial neural networks can harness stochasticity in multiple ways to enable a vast class of computationally powerful models. Boltzmann machines and other stochastic neural networks have been shown to outperform their deterministic counterparts by allowing dynamical systems to escape local energy minima. Electronic implementation of such stochastic networks is currently limited to addition of algorithmic noise to digital machines which is inherently inefficient; albeit recent efforts to harness physical noise in devices for stochasticity have shown promise. To succeed in fabricating electronic neuromorphic networks we need experimental evidence of devices with measurable and controllable stochasticity which is complemented with the development of reliable statistical models of such observed stochasticity. Current research literature has sparse evidence of the former and a complete lack of the latter. This motivates the current article where we demonstrate a stochastic neuron using an insulator-metal-transition (IMT) device, based on electrically induced phase-transition, in series with a tunable resistance. We show that an IMT neuron has dynamics similar to a piecewise linear FitzHugh-Nagumo (FHN) neuron and incorporates all characteristics of a spiking neuron in the device phenomena. We experimentally demonstrate spontaneous stochastic spiking along with electrically controllable firing probabilities using Vanadium Dioxide (VO_2_) based IMT neurons which show a sigmoid-like transfer function. The stochastic spiking is explained by two noise sources - thermal noise and threshold fluctuations, which act as precursors of bifurcation. As such, the IMT neuron is modeled as an Ornstein-Uhlenbeck (OU) process with a fluctuating boundary resulting in transfer curves that closely match experiments. The moments of interspike intervals are calculated analytically by extending the first-passage-time (FPT) models for Ornstein-Uhlenbeck (OU) process to include a fluctuating boundary. We find that the coefficient of variation of interspike intervals depend on the relative proportion of thermal and threshold noise, where threshold noise is the dominant source in the current experimental demonstrations. As one of the first comprehensive studies of a stochastic neuron hardware and its statistical properties, this article would enable efficient implementation of a large class of neuro-mimetic networks and algorithms.

## 1. Introduction

A growing need for efficient machine-learning in autonomous systems coupled with an interest in solving computationally hard optimization problems has led to active research in stochastic models of computing. Optimization techniques (Haykin, 2009) including Stochastic Sampling Machines (SSM), Simulated Annealing, Stochastic Gradients etc., are examples of such models. All these algorithms are currently implemented using digital hardware which first creates a mathematically accurate platform for computing, and later adds digital noise at the algorithm level. Hence, it is enticing to construct hardware primitives that can harness the already existing physical sources of noise to create a stochastic computing platform. The principal challenge with such efforts is the lack of stable or reproducible distributions, or functions of distributions, of physical noise. One basic stochastic unit which enables a systematic construction of stochastic hardware has long been known—the stochastic neuron (Gerstner and Kistler, 2002)—which is also believed to be the unit of computation in the human brain. Moreover, recent studies (Buesing et al., 2011) have demonstrated practical applications like sampling using networks of such stochastic spiking neurons. There have been some attempts for building neuron hardware (Indiveri et al., 2006; Pickett et al., 2013; Mehonic and Kenyon, 2016; Sengupta et al., 2016; Tuma et al., 2016), but building a neuron with self-sustained spikes, or oscillations, which are stochastic in nature and where the probability of firing is controllable using a signal has been challenging. Here, we demonstrate and analytically study a true stochastic neuron (Jerry et al., 2017a) which is fabricated using oscillators (Shukla et al., 2014a,b; Parihar et al., 2015) based on insulator-metal transition (IMT) materials, e.g., Vanadium Dioxide (VO_2_), wherein the inherent physical noise in the dynamics is used to implement stochasticity. The firing probability, and not just the deterministic frequency of oscillations or spikes, is controllable using an electrical signal. We also show that such an IMT neuron has similar dynamics as a piecewise linear FitzHugh-Nagumo (FHN) neuron with thermal noise along with threshold fluctuations as precursors of bifurcation resulting in a sigmoid-like transfer function for the neural firing rates. By analyzing the variance of interspike interval, we determine that for the range of thermal noise present in our experimental demonstrations, threshold fluctuations are responsible for most of the stochasticity compared to thermal noise.

## 2. Materials and methods

### 2.1. IMT phase change neuron model

**Figure 1:**
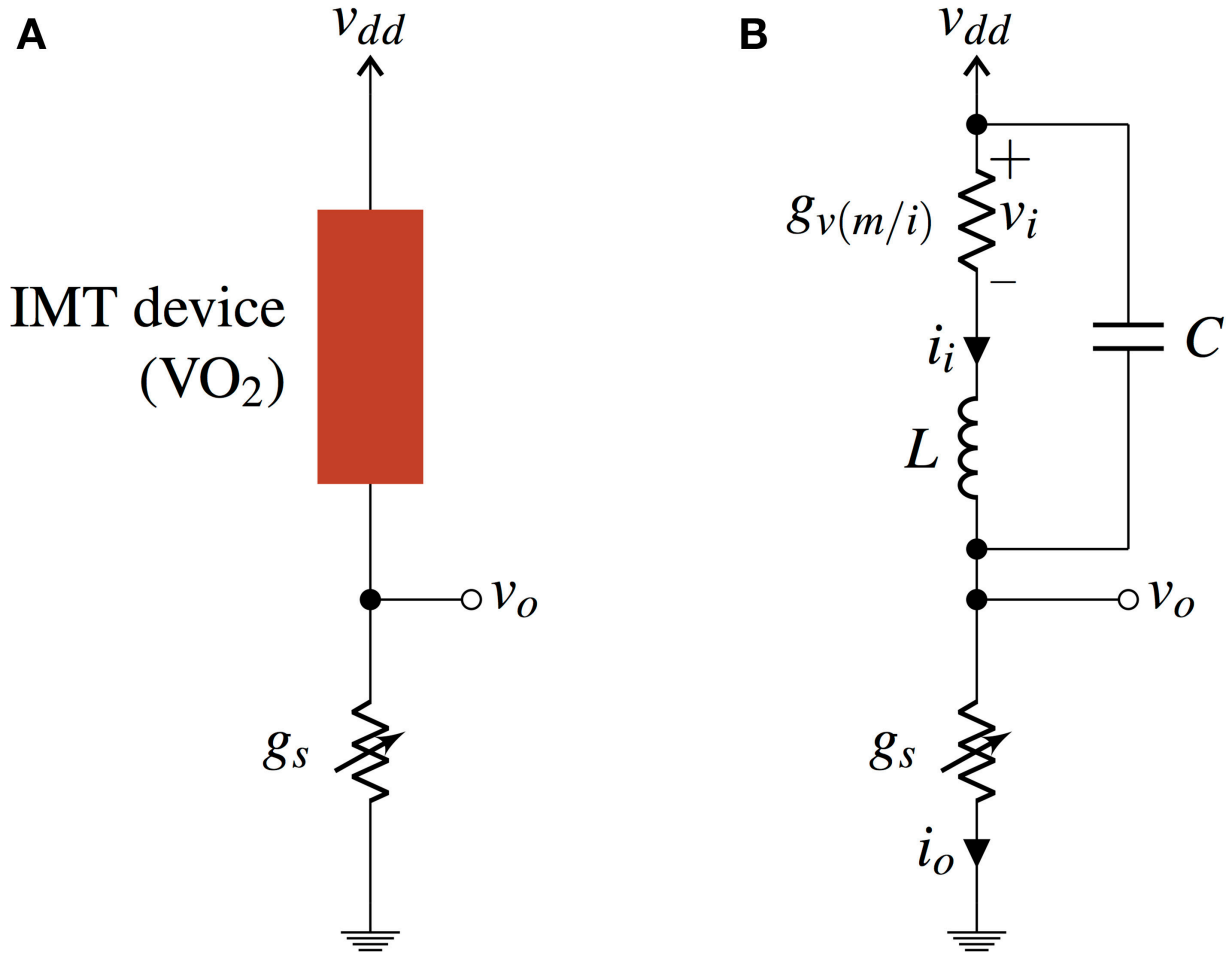
**(A)** VO_2_ based IMT spiking neuron circuit consisting of a VO_2_ device in series with a tunable resistance. **(B)** Equivalent circuit of IMT neuron using a series inductance *L* and a parallel capacitance *C*.

A stochastic IMT neuron is fabricated using relaxation oscillators (Shukla et al., 2014b; Parihar et al., 2015) composed of an IMT phase change device, e.g., Vanadium Dioxide (VO_2_), in series with a tunable resistance, e.g., transistor (Shukla et al., 2014a) (Figure 1A). An IMT device is a two terminal device with two resistive states—insulating (I) and metallic (M), and the device transitions between the two states based on the applied electric field (which in turn changes the current through the device and the corresponding temperature) across it. The phase transitions are hysteretic in nature, which means that the IMT (insulator-to-metal) transition does not occur at the same voltage as the MIT (metal-to-insulator) transition. For a range of values of the series resistance, the resultant circuit shows spontaneous oscillations due to hysteresis and a lack of stable point (Parihar et al., 2015). Overall, the series resistance acts as a parameter for bifurcation between a spiking (or oscillating) state and a resting state of an IMT neuron.

The equivalent circuit model for an IMT oscillator is shown in Figure 1B with the hysteretic switching conductance *g*_*v*(*m*/*i*)_ (*g*_*vm*_ in metallic and *g*_*vi*_ in insulating state), a series inductance *L*, and a parallel internal capacitance *C*. Let the IMT and MIT thresholds of the device be denoted by *v*_*h*_ and *v*_*l*_, respectively, with *v*_*h*_ > *v*_*l*_, and the current-voltage relationship of the hysteretic conductance be

$$v_{i}=h(i_{i},s)$$

where *h* is linear in *i*_*i*_ and *s* is the state—metallic (M) or insulating (I).

The system dynamics is then given by:

(1) $$\begin{array}{l} \;L\frac{di_{i}}{dt}=(v_{dd}-h(i_{i},s))-v_{o}\\ C\frac{dv_{o}}{dt}=i_{i}-g_{s}v_{o} \end{array}$$

with *i*_*i*_ and *v*_*o*_ as shown in Figure 1B and *s* is considered as an independent variable.

### 2.2. Mechanism of oscillations and spikes

**Figure 2:**
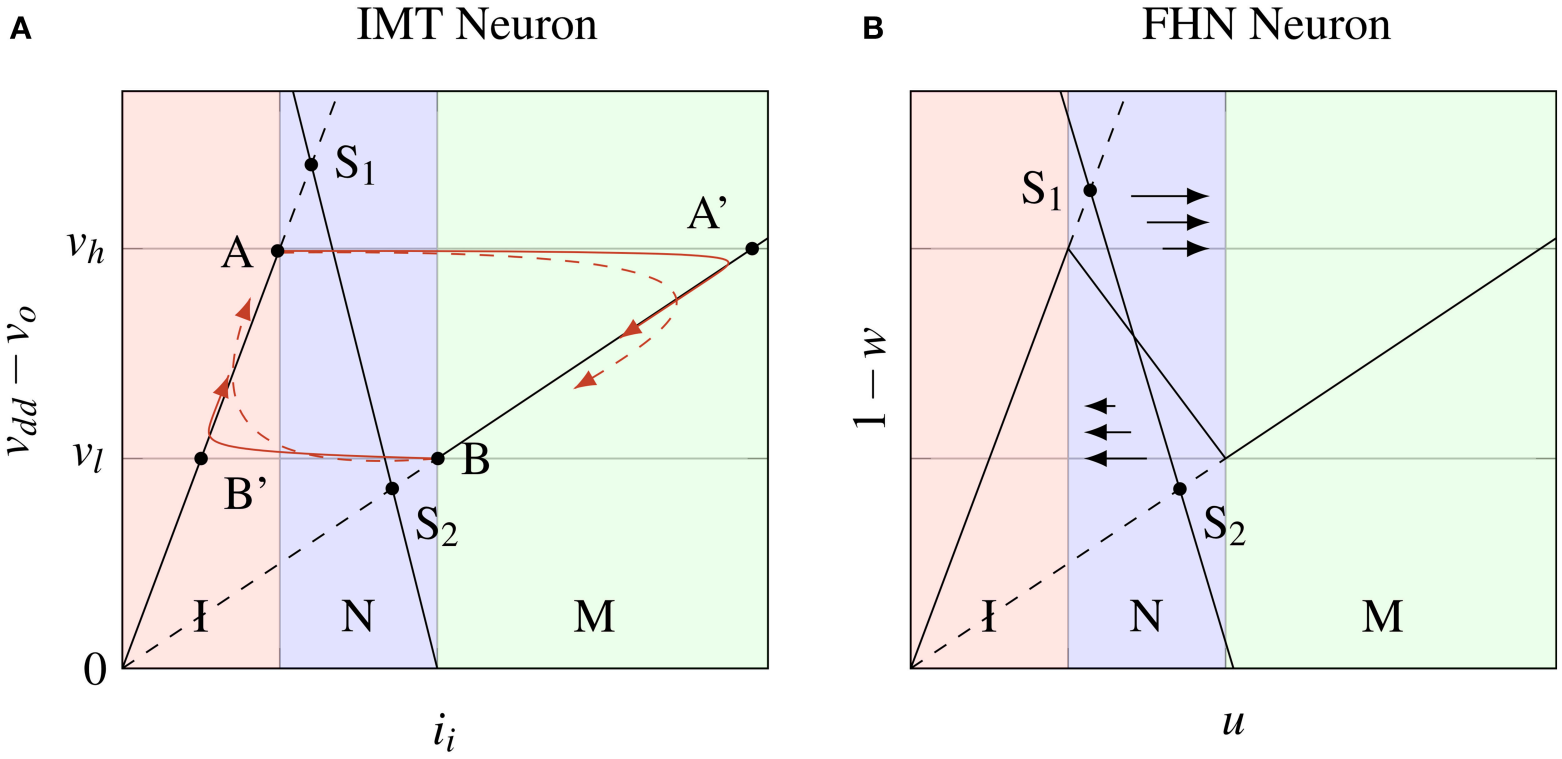
**(A)** Trajectories (red) of system (1) in the phase space *i*_*i*_ × (*v*_*dd*_ − *v*_*o*_) for a small *L*/*C* value (solid) and a large *L*/*C* value (dashed). The *i*_*i*_-nullclines of system (1) are shown as solid black lines in the metallic (AB') and insulating (BA') states of the IMT device, and S_1_S_2_ is the *v*_*o*_-nullcline. Depending on the state, the phase space is divided into three vertical regions - I, M and N. In the region N the *i*_*i*_-nullclines are dependent on *s* **(B)** Nullclines of the FHN model in the phase space *u* × (1 − *w*) where *f*(*u*) is a piecewise linear function. The dynamics of FHN neuron are equivalent to the IMT neuron in the regions M and I. In the region N, for small *L*/*C*, the difference is only in the velocity and not the direction of system trajectories as they are parallel to *x*-axis.

In VO_2_, IMT, and MIT transitions are orders of magnitude faster than RC time constants for oscillations, as observed in frequency (Kar et al., 2013) and time-domain measurements for voltage driven (Jerry et al., 2016) and photoinduced transitions (Cocker et al., 2012). As such, the change in resistance of the IMT device is assumed to be instantaneous. Figure 2A shows the phase space *i*_*i*_ × (*v*_*dd*_ − *v*_*o*_). V-I curves for IMT device in the two states metallic (M) and insulating (I) and the load line for series conductance *v*_*o*_ = *i*_*i*_/*g*_*s*_ for the steady state are shown along with the fixed points of the system *S*_1_ and *S*_2_ in insulating and metallic states respectively. The load line and V-I curves are essentially the nullclines of *v*_*o*_ and *i*_*i*_, respectively. The capacitance- inductance pair delays the transitions and slowly pulls the system toward the fixed points S_1_ and S_2_ even when the IMT device transitions instantaneously. For small *L*/*C* ratio, the eigenvector (of the coefficient matrix) with large negative eigenvalue becomes parallel to the x-axis, whereas the other eigenvector becomes parallel to AB′ or BA′ depending on the state (M or I). When the system approaches A from below (or B from above) and IMT device is insulating (or metallic) with fixed point *S*_1_ (or *S*_2_), the IMT device transitions into metallic (or insulating) state changing the fixed point to S_2_ (or *S*_1_). Two trajectories are shown starting from points A and B each for the system (Equation 1)—one for small *L*/*C* value (solid) and the other for large *L*/*C* value (dashed). After a transition, the system moves parallel to *x*-axis almost instantaneously and spends most of the time following the V-I curve toward the fixed point. Before the fixed point is reached the MIT (or IMT) transition threshold is encountered which switches the fixed point, and the cycle continues resulting in sustained oscillations or spike generation.

### 2.3. Model approximations and connections with FHN neuron

#### 2.3.1. Non-hysteretic approximation

**Figure 3:**
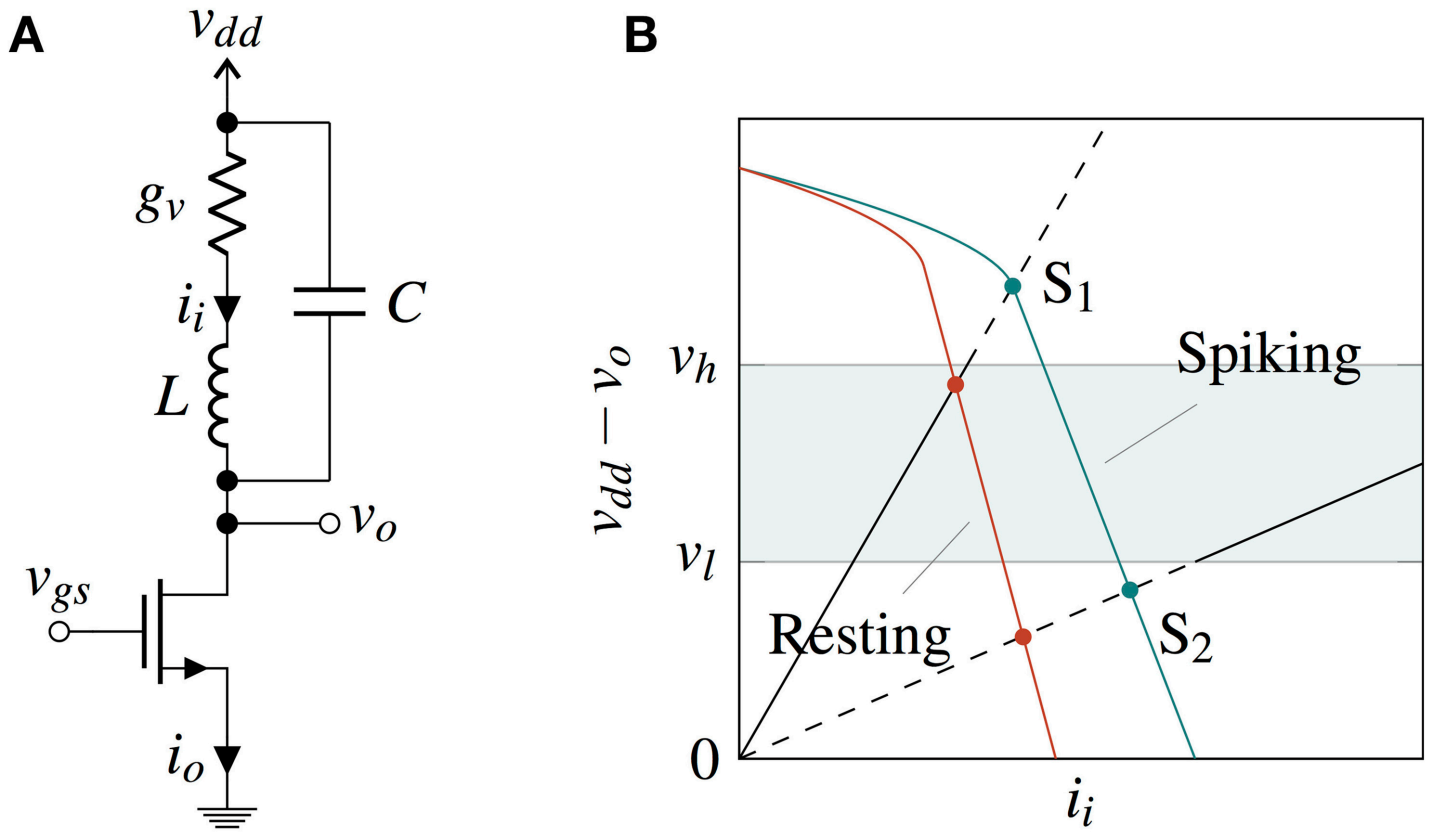
**(A)** IMT neuron with series transistor used to achieve bifurcation between a spiking and a resting state. **(B)** Nullclines of the system with series transistor in the phase space *i*_*i*_ × *v*_*dd*_ − *v*_*o*_ for two different *v*_*gs*_ values for spiking and resting states. Bifurcation occurs when a stable points crosses the boundary of region *v*_*dd*_ − *v*_*o*_ ∈ [*v*_*l*_, *v*_*h*_].

The model of (Equation 1) is very similar to a piecewise linear caricature of FitzHugh-Nagumo (FHN) neuron model (Gerstner and Kistler, 2002), also called the McKean's caricature (McKean, 1970; Tonnelier, 2003). Mathematically, the FHN model is given by:

(2) $$\begin{array}{l} \;\frac{du}{dt}=f(u)-w+I_{ext}\\ \tau \frac{dw}{dt}=u-bw+a \end{array}$$

where *f*(*u*) is a polynomial of third degree, e.g., *f*(*u*) = *u* − *u*^3^/3, and *I*_*ext*_ is the parameter for bifurcation, as opposed to *g*_*s*_ in Equation (1). In the FHN model, one variable (*u*), possessing cubic nonlinearity, allows regenerative self-excitation via a positive feedback, and the second, a recovery variable (*w*), possessing linear dynamics, provides a slower negative feedback. It was reasoned in McKean (1970) that the essential features of FHN model are retained in a “caricature” where the cubic non-linearity is replaced by a piecewise linear function *f*(*u*). Nullclines of (Equation 2) with a piecewise linear *f*(*u*) are shown in Figure 2B in the phase space *u* × (1 − *w*). A function *f*(*u*) is trivially possible such that it is equal to *v*_*dd*_ − *h*(*i*_*i*_, *s*) in the regions M and I, hence making the *u*-nullcline similar to the *i*_*i*_-nullcline in those regions. In the region N, the difference between *f*(*u*) and *v*_*dd*_ − *h*(*i*_*i*_, *s*) for any state *s* does not result in a difference in the direction of system trajectories but only in their velocity, because for small *L*/*C* the trajectories are almost parallel to *x*-axis. Bifurcation in VO_2_ neuron is achieved by tuning the load line using a tunable resistance (*g*_*s*_), or a series transistor (Figure 3A). Figure 3B shows two load line curves corresponding to different gate voltages (*v*_*gs*_), where one gives rise to spikes while the other results in a resting state.

#### 2.3.2. Single dimensional approximation

**Figure 4:**
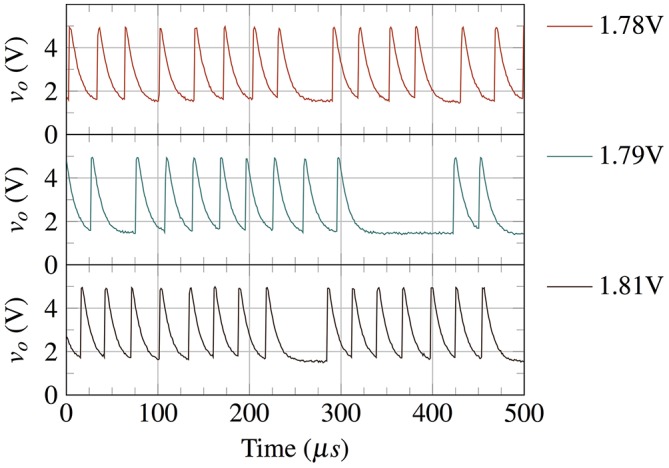
Experimental waveforms of VO_2_ based spiking neuron for various *v*_*gs*_ values (1.78, 1.79, and 1.81 V). A VO_2_ neuron shows almost instantaneous charging (spike) in metallic state.

Moreover, a single dimensional piecewise approximation of the system can be performed using a dimensionality reduction by replacing the movement along the eigenvector parallel to the x-axis with an instantaneous transition from A to A′, or B to B′. This leaves a 1-dimensional subsystem in M and I each along the V-I curves AB′ and BA′. Experiments using VO_2_ show that the metallic state conductance *g*_*vm*_ is very high which causes the charging cycle of *v*_*o*_ to be almost instantaneous (Figure 4) and resembles a spike of a biological neuron. As such, the spiking statistics can be studied by modeling just the discharge cycle of *v*_*o*_. The inductance being negligible can be effectively removed and only the capacitance is needed for modeling the 1D subsystem of insulating state (**Figure 6A**) making *v*_*i*_ = *v*_*dd*_ − *v*_*o*_.

### 2.4. Noise induced stochastic behavior

The two important noise sources which induce stochasticity in an IMT neuron are (a) V_*IMT*_ (*v*_*h*_) fluctuations (Zhang et al., 2016; Jerry et al., 2017b), and (b) thermal noise. Thermal noise η(*t*) is modeled in the circuit (**Figure 6A**) as a white noise voltage η(*t*)d*t* = σ_*t*_d*w*_*t*_ where *w*_*t*_ is the standard weiner process and $\sigma_{t}^{2}$ is the infinitesimal thermal noise variance. The threshold *v*_*h*_ is assumed constant during a spike, but varies from one spike to another. The distribution of *v*_*h*_ from spike to spike is assumed to be Gaussian or subGaussian whose parameters are estimated from experimental observations of oscillations. If the series transistor always remains in saturation and show linear voltage-current relationship, as is the case in our VO_2_ based experiments, the discharge phase can be described by an Ornstein-Uhlenbeck (OU) process

(3) $$\text{d}x=\frac{1}{\theta }(\mu -x)\text{d}t+\sigma \text{d}w_{t}$$

where μ, θ, and σ are functions of circuit parameters of the series transistor, the IMT device and σ_*t*_. The interspike interval is thus the first-passage-time (FPT) of this OU process, but with a fluctuating boundary.

#### 2.4.1. OU process with constant boundary

Analytical expressions for the FPT of OU process (with μ = 0) for a constant boundary were derived using the Laplace transform method in Ricciardi and Sato (1988). Reproducing some of its results, let the first passage time for the system (Equation 3), with μ = 0, which starts at *x*(0) = *x*_0_ and hits a boundary *S*, be denoted by the random variable **t_f_**(*S, x*_0_), and its *m*th moment by τ_*m*_(*S, x*_0_). Also, let $\tilde{t_{f}}(S,x_{0})$ be the FPT for another OU process with μ = 0, θ = 1, and σ = 2, and $\tilde{\tau_{m}}\left(S,x_{0}\right)$ be its *mth* moment. Then time and space scaling for the OU process imply that

(4) $$\begin{array}{l} \;t_{f}(S,x_{0})\overset{d}{=}\theta \tilde{t_{f}}(\alpha S,\alpha x_{0})\\ ∴\tau_{m}(S,x_{0})=\theta^{m}\tilde{\tau_{m}}(\alpha S,\alpha x_{0}) \end{array}$$

where $\alpha =\sqrt{\frac{2}{\theta \sigma^{2}}}$. The first two moments for the base case OU process $\tilde{\tau_{1}}$ and $\tilde{\tau_{2}}$ are given by

(5) $$\begin{array}{l} \tilde{\tau_{1}}(S,x_{0})=\phi_{1}(S)-\phi_{1}(x_{0})\\ \tilde{\tau_{2}}(S,x_{0})=2\phi_{1}{\left(S\right)}^{2}-\phi_{2}(S)-2\phi_{1}(S)\phi_{1}(x_{0})+\phi_{2}(x_{0}) \end{array}$$

where ϕ_*k*_(*z*) can be written as an infinite sum

(6) $$\phi_{k}(z)=\frac{1}{2^{k}}\sum_{n=1}^{\infty }\frac{{\left(\sqrt{2}z\right)}^{n}\Gamma \left(\frac{n}{2}\right)\rho (n,k)}{n!}$$

with ρ(*n, k*) being a function of the digamma function (Ricciardi and Sato, 1988).

#### 2.4.2. OU process with fluctuating boundary

We extend this framework for calculating the FPT statistics with a fluctuating boundary **S** as follows. Let the IMT threshold be represented by the random variable **v_h_**. For the VO_2_ based IMT neuron, the 1D subsystem in the insulating phase can be converted in the form of Equation(3) with μ = 0 by translating the origin to the fixed point. If this transformation is **T** then *x* = **T***v*_*i*_ = **T**(*v*_*dd*_ − *v*_*o*_), **S** = **Tv_h_**, and *x*_*o*_ = **T***v*_*l*_. The start and end points are B′ and A, respectively in Figure 2. **v_h_** is assumed constant during a spike, and across spikes the distribution of **v_h_** is **v_h_** ~ D, where D is either Gaussian, or subGaussian. For subGaussian distributions we use the Exponential Power family EP[κ], κ being the shape factor. Let the interspike interval of IMT neuron be denoted by the marginal random variable $t_{imt}(D,v_{l})$. Then **t_imt_** is related to **t_f_** in Equation (4), given common parameters θ and σ, as follows:

$$t_{imt}(D,v_{l})|(v_{h}=v)\overset{d}{=}t_{f}(Tv,Tv_{l})$$

The moments of **t_imt_** can be calculated as:

(7) $$\begin{array}{l} 𝔼\left[t_{imt}{\left(D,v_{l}\right)}^{m}\right]={𝔼}_{v_{h}}\left[𝔼\left[t_{imt}{\left(D,\text{T}v_{l}\right)}^{m}|v_{h}=v\right]\right]\\ \;={𝔼}_{v_{h}}[\tau_{m}(Tv_{h},Tv_{l})]\\ \;=\theta^{m}{𝔼}_{v_{h}}[\tilde{\tau_{m}}(\alpha Tv_{h},\alpha Tv_{l})] \end{array}$$

where $\alpha =\sqrt{\frac{2}{\theta \sigma^{2}}}$. If D is Gaussian or EP[κ] distribution and α**T** is an affine transformation, then α**Tv_h_** also has a Gaussian or EP[κ] distribution.

### 2.5. Experiments

IMT devices are fabricated on a 10nm VO_2_ thin film grown by reactive oxide molecular beam epitaxy on (001) TiO_2_ substrate using a Veeco Gen10 system (Tashman et al., 2014). Planar two terminal structures are formed by patterning contacts using standard electron beam lithography which defines the device length (L_*VO*2_). Pd (20 nm)/Au (60 nm) contacts are then deposited by electron beam evaporation and liftoff. The devices are then isolated and the widths (W_*VO*2_) are defined using a CF_4_ based dry etch.

**Figure 5:**
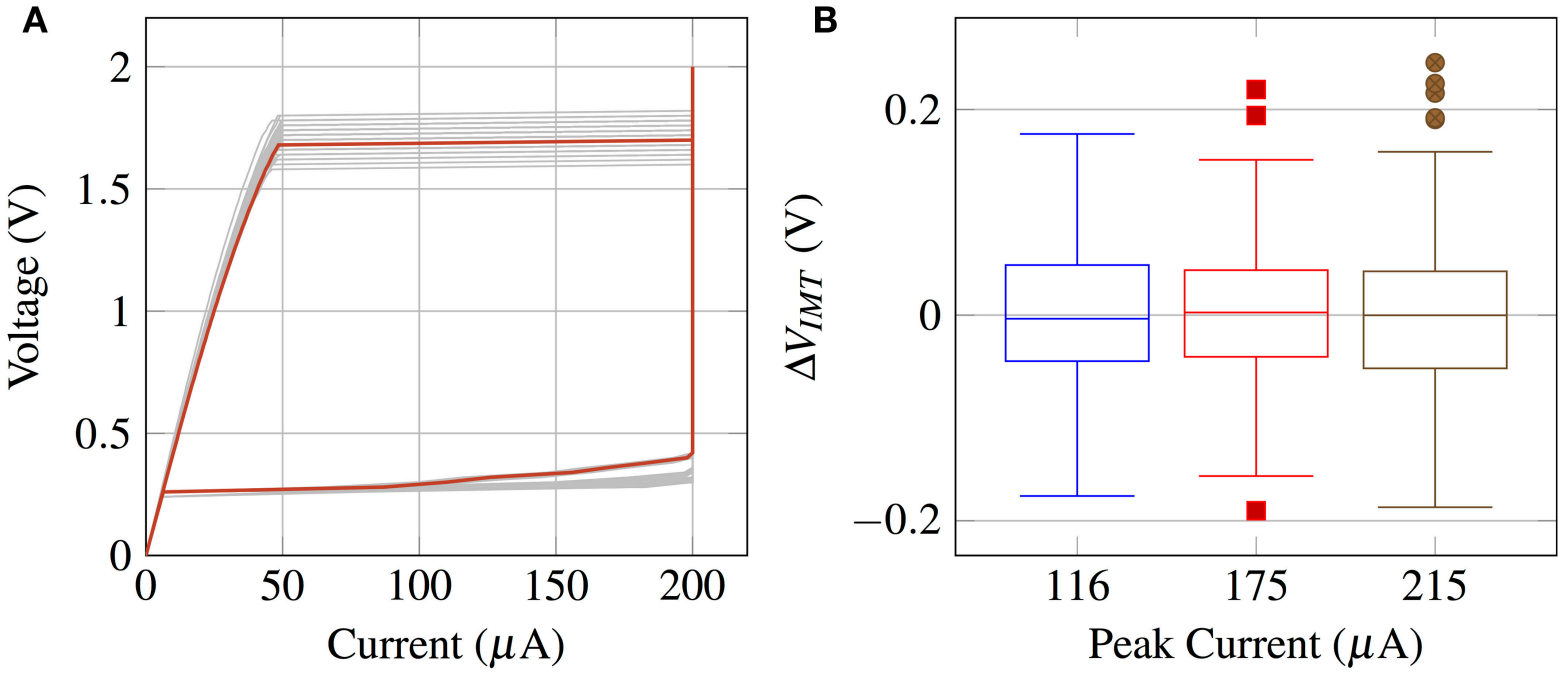
**(A)** The prototypical DC voltage-current characteristics for a single VO_2_ device exhibits abrupt threshold switching at V_*IMT*_ and V_*MIT*_. The current in the metallic state has been arbitrarily limited to a 200μA compliance current. **(B)** V_*IMT*_ distribution as a function of the peak current during oscillations (value is set by the MOSFET saturation current). V_*IMT*_ is extracted from 300+ cycles.

The IMT neuron is constructed using an externally connected n-channel MOSFET (ALD110802) and the fabricated VO_2_ device. A prototypical I-V curve is shown in Figure 5A. Within the experimental data, the current is limited to an arbitrarily chosen 200 μA to prevent a thermal runaway and breakdown of the device while in the low resistance metallic state. It should be noted that as the metallic state corresponds to the abrupt charging cycle of *v*_*o*_, limiting the current would not have noticeable effect on spiking statistics of the neuron.

**Figure 6:**
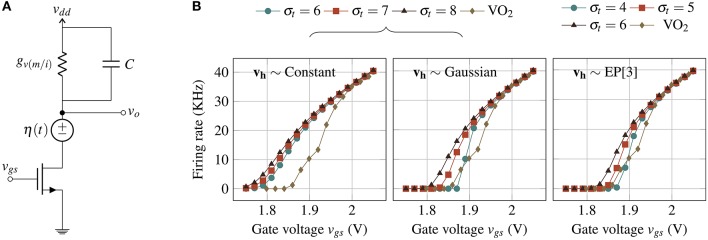
**(A)** Noise model of IMT neuron where the noise components are the thermal noise voltage source η(*t*) and the IMT threshold fluctuation. **(B)** Firing rate plotted against *v*_*gs*_ using the analytical model for different **v_h_** distributions (Constant, Gaussian, and EP[3]) and comparison with experimental observations.

Threshold voltage fluctuations (cycle to cycle) were observed in all devices which were tested (>10). Threshold voltage distribution was estimated using the varying cycle-to-cycle threshold voltages collected from a single device. Thermal noise is not measured directly, but is estimated approximately by matching the simulation waveforms from the circuit model (Figure 6A) with the observed experimental waveforms. It can be verified that thermal noise of the transistor is not the dominant noise source by measuring the threshold variation as a function of the transistor current (Figure 5B) and observing that the distribution of switching threshold does not change with varying transistor current. Finally, the firing rate and its variation with *v*_*gs*_ (Figure 6B) were measured for a single device.

## 3. Results

### 3.1. Spiking statistics

#### 3.1.1. First moment and the firing rate

First moment of **t_imt_** is calculated using Equations (5) and (7) as

$$𝔼[t_{imt}(D,v_{l})]=\theta ({𝔼}_{v_{h}}[\phi_{1}(\alpha Tv_{h})]-\phi_{1}(\alpha x_{0}))$$

The expansion for ϕ_*k*_(*z*) in Equation(6) can be used to calculate 𝔼_*v*_*h*__[ϕ_*k*_(α**Tv_h_**)] using the moments of α**Tv_h_** as follows

$${𝔼}_{v_{h}}[\phi_{k}(\alpha Tv_{h})]=\frac{1}{2^{k}}\sum_{n=1}^{\infty }\frac{{\left(\sqrt{2}\right)}^{n}𝔼[{\left(\alpha Tv_{h}\right)}^{n}]\Gamma \left(\frac{n}{2}\right)\rho (n,k)}{n!}$$

Figure 6B shows firing rate ($1/𝔼[t_{imt}(D,v_{l})]$) as a function of *v*_*gs*_ for various σ_*t*_ values and for three distributions of threshold fluctuations. The calculations approximate the experimental observations well for all three *v*_*h*_ distributions, the closest being EP[3] with σ_*t*_ = 4.

#### 3.1.2. Higher moments

For higher moments, higher order terms are encountered. For example, in case of the second moment, using Equations(5) and (7), we obtain

$$\begin{array}{l} {𝔼}_{v_{h}}[\tilde{\tau_{2}}(\alpha Tv_{h},\alpha Tv_{l})]=\;2{𝔼}_{v_{h}}\left[\phi_{1}{\left(\alpha Tv_{h}\right)}^{2}\right]-{𝔼}_{v_{h}}\left[\phi_{2}\left(\alpha Tv_{h}\right)\right]\\ \;-2{𝔼}_{v_{h}}[\phi_{1}(\alpha Tv_{h})]\phi_{1}(\alpha Tv_{l})\\ \;+\phi_{2}(\alpha Tv_{l}) \end{array}$$

with a higher order term $\phi_{1}{\left(\alpha Tv_{h}\right)}^{2}$. In the case of the third moment we obtain ϕ_1_(α**Tv_h_**)ϕ_2_(α**Tv_h_**). As each ϕ_*k*_ term is an infinite sum, we construct a cauchy product expansion for the higher order term using the infinite sum expansions of the constituent ϕ_*k*_s and then distribute the expectation over addition. For example, if the ϕ_*k*_ expansions of ϕ_1_(*z*) and ϕ_2_(*z*) are (∑*a*_*i*_) and (∑*b*_*i*_), respectively, then the cauchy product expansion of ϕ_1_(*z*)ϕ_2_(*z*) can be calculated as ∑*c*_*i*_, where *c*_*i*_ is a function of *a*_1…*i*_ and *b*_1…*i*_, and the expectation 𝔼[ϕ_1_(*z*)ϕ_2_(*z*)] = ∑𝔼[*c*_*i*_]. Since *c*_*i*_ is a polynomial in *z*, 𝔼[*c*_*i*_] can be calculated using the moments of *z*.

**Figure 7:**
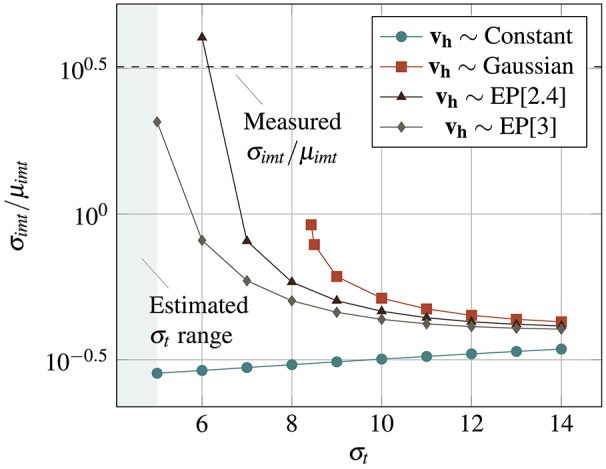
σ_*imt*_/μ_*imt*_ for the interspike interval plotted against σ_*t*_ for *v*_*gs*_ = 1.8V with Constant, Gaussian, and Exponential Power (EP[κ], where κ is the shape factor) distributions of the threshold noise. The experimentally observed σ_*imt*_/μ_*imt*_ for a VO_2_ neuron is shown with a dotted line. The shaded region shows the experimentally estimated range of σ_*t*_ (σ_*t*_ < 5).

If μ_*imt*_ and σ_*imt*_ are the mean and standard deviation of interspike intervals **t_imt_**, the coefficient of variation (σ_*imt*_/μ_*imt*_) varies with the relative proportion of the thermal and the threshold induced noise. Figure 7 shows σ_*imt*_/μ_*imt*_ (calculated using parameters matched with our VO_2_ experiments) plotted against σ_*t*_ for various kinds of **v_h_** distributions fitted to experimental observations. σ_*imt*_/μ_*imt*_ as observed in our VO_2_ experiments is about an order of magnitude more than what would be calculated with only thermal noise using such a neuron, and hence, threshold noise contributes significant stochasticity to the spiking behavior. As the IMT neuron is setup such that the stable point is close to the IMT transition point (Figure 3B), low σ_*t*_ results in high and diverging σ_*imt*_/μ_*imt*_ for any distribution of threshold noise, and σ_*imt*_/μ_*imt*_ reduces with increasing σ_*t*_ for the range shown. For a Normally distributed *v*_*h*_ the variance diverges for σ_*t*_ ≲ 8, but for Exponential Power (EP) distributions with lighter tails, the variance converges for smaller values of σ_*t*_. Statistical measurements on experimental data, as indicated in Figure 7, provide measures of σ_*imt*_/μ_*imt*_ (dotted line) and σ_*t*_ (shaded region). We note that EP distributions provide a better approximation of the stochastic nature of experimentally demonstrated VO_2_ neurons as the range of σ_*t*_ is estimated to be <5.

## 4. Discussion

In this paper, we demonstrate and analyse an IMT based stochastic neuron hardware which relies on both threshold fluctuations and thermal noise as precursors to bifurcation. The IMT neuron emulates the functionality of theoretical neuron models completely by incorporating all neuron characteristics into device phenomena. Unlike other similar efforts, it does not need peripheral circuits alongside the core device circuit (an IMT device and a transistor) to emulate any sub-component of the spiking neuron model like thresholding, reset etc. Moreover, the neuron construction not only utilizes inherent physical noise sources for stochasticity, but also enables control of firing probability using an analog electrical signal—the gate voltage of series transistor. This is different from previous works which control only the deterministic aspect of firing rate like the charging rate. A comparison of spiking neuron hardware characteristics in different works is shown in Table 1.

**Table 1:** Comparison of this work (experimental details from Jerry et al., 2017a) with other spiking neuron hardware works based on different characteristics of spiking neurons.

	**Tuma et al., 2016**	**Pickett et al., 2013**	**Sengupta et al., 2016**	**Indiveri et al., 2006**	**This work (VO_2_)**
Neuron type	Integrate & Fire	Hodgkin Huxley	Integrate & Fire	Integrate & Fire	Piecewise Linear FHN
Material/Platform	Chalcogenide	Mott insulator NbO_2_	MTJ	0.35 μm CMOS	Vanadium Dioxide (VO_2_)
Material phenomenon	Phase Change	IMT	Spin transfer torque (STT)	–	IMT
Spontaneous spiking using only device	No	Yes	No	–	Yes
Peripherals needed for spiking	Yes, for spike generation and reset	No	Yes, for spike generation and reset	–	No
Integration mechanism (I&F)	Heat accumulation	–	Magnetization accumulation	Capacitor charging	Capacitor charging
Threshold mechanism (I&F)	External reset by measuring conductance	Spontaneous IMT	External reset by detecting magnet flip	Reset using comparator	Spontaneous IMT
Stochastic	Yes	–	Yes	No	Yes
Kind of stochasticity (I&F)	Reset potential	–	Differential	–	Threshold and differential
Source of stochasticity / noise	Melt-quench process	–	Thermal noise	–	IMT threshold fluctuations & Thermal noise
Control of stochastic firing rate	Only integration rate	–	Only integration rate	Only integration rate	Yes
Status of experiments	Constant stochasticity, variable integration rate	Deterministic spiking	None	Deterministic spiking	Sigmoidal variation of stochastic firing rates
Peak current	750–800 μA		–		200 μA
Power or Energy/spike	120 μW		–	900 pJ / spike	196 pJ / spike
Voltage	5.5 V	1.75 V	–	3.3 V	0.7 V
Maximum firing rates	35–40 KHz	30 KHz	–	200 Hz	30 KHz

We also show that the neuron dynamics follow a linear “carricature” of the FitzHugh-Nagumo model with intrinsic stochasticity. The analytical models developed in this paper can also faithfully reproduce the experimentally observed transfer curve which is a stochastic property. Such analytical verification of stochastic neuron experiments is one of the first in this work. It is an important result as it indicates reproducibility of stochastic characteristics and helps in creating the pathway toward perfecting these devices. With a growing concensus that stochasticity will play a key role in solving hard computing tasks, we need efficient ways for controlled amplification and conversion of physical noise into a readable and computable form. In this regard, the IMT based neuron represents a promising solution for a stochastic computational element. Such stochastic neurons have the potential to realize bio-mimetic computational kernels that can be employed to solve a large class of optimization and machine-learning problems.

## Author contributions

AP worked on the development of theory, simulation frameworks, and mathematical models; MJ worked on the experiments; AR advised AP and participated in the problem formulation; SD advised MJ and also participated in the design of experiments and problem formulations.

### Conflict of interest statement

The authors declare that the research was conducted in the absence of any commercial or financial relationships that could be construed as a potential conflict of interest.
